# Childcare as a social determinant of access to healthcare: a scoping review

**DOI:** 10.3389/fpubh.2024.1443992

**Published:** 2024-12-03

**Authors:** Megan McArthur, Persephone Tian, Kimberly A. Kho, Kavita P. Bhavan, Bijal A. Balasubramanian, Anisha P. Ganguly

**Affiliations:** ^1^University of Texas Southwestern Medical School, University of Texas Southwestern Medical Center, Dallas, TX, United States; ^2^Division of Gynecology, Department of Obstetrics and Gynecology, University of Texas Southwestern Medical Center, Dallas, TX, United States; ^3^Center of Innovation and Value at Parkland, Parkland Health, Dallas, TX, United States; ^4^Division of Infectious Diseases, Department of Internal Medicine, University of Texas Southwestern Medical Center, Dallas, TX, United States; ^5^Department of Epidemiology, Human Genetics, and Environmental Sciences, School of Public Health, University of Texas Health Science Center at Houston, Houston, TX, United States; ^6^Institute for Implementation Science, School of Public Health, University of Texas Health Science Center at Houston, Houston, TX, United States; ^7^Division of General Medicine and Clinical Epidemiology, Department of Internal Medicine, University of North Carolina at Chapel Hill, Chapel Hill, NC, United States

**Keywords:** childcare, social determinants of health, access to healthcare, caregiver, health-related social need, health equity

## Abstract

**Introduction:**

As health systems strive to screen for and address social determinants of health (SDOH), the role of access to childcare and barriers to healthcare posed by childcare needs remains underexplored. A gap exists in synthesizing existing evidence on the role of access to childcare as a SDOH.

**Methods:**

This scoping review aimed to examine and analyze existing literature on the role of childcare needs as a social determinant of access to healthcare. We conducted a structured literature search across PubMed, Scopus, health policy fora, and professional healthcare societies to inclusively aggregate studies across interdisciplinary sources published between January 2000 and June 2023. Two independent reviewers reviewed results to determine inclusions and exclusions. Studies were coded into salient themes utilizing an iterative inductive approach.

**Results:**

Among 535 search results, 526 met criteria for eligibility screening. Among 526 eligible studies, 91 studies met inclusion criteria for analysis. Five key themes were identified through data analysis: (1) barriers posed by childcare needs to healthcare appointments, (2) the opportunity for alternative care delivery models to overcome childcare barriers, (3) the effect of childcare needs on participation in medical research, (4) the impact of the COVID-19 pandemic on childcare needs, and (5) the disproportionate burden of childcare experienced by vulnerable populations.

**Discussion:**

Childcare needs remain underexplored in existing research. Current evidence demonstrates the relevance of childcare needs as a barrier to healthcare access, however dedicated studies are lacking. Future research is needed to understand mechanisms of childcare barriers in access to healthcare and explore potential interventions.

## 1 Introduction

Social determinants of health (SDOH) like housing, food security, transportation, and insurance, are the non-medical conditions that drive health outcomes (1). Access to healthcare, defined as all factors that facilitate or impede the use of healthcare services (2), cannot be viewed simply through the lens of availability of services, but also the means to obtain and utilize services (3). Recognizing that SDOH inequitably impact marginalized and minoritized communities, we must understand and address social and structural factors affecting engagement in healthcare to address health disparities (4, 5).

Recent analyses of barriers to care engagement in myriad clinical contexts demonstrate the importance of health-related social needs (HRSN) that affect presentation to care and adherence (6–9). Transportation (10, 11) and work leave (12, 13) have been established as HRSNs that are associated with missed appointments, deferral of care, and medication non-adherence (14, 15). Similarly, childcare needs are increasingly recognized as a driver of missed and deferred care, as notably highlighted by the 2017 Kaiser Family Foundation Women’s Health Survey (16). Childcare has emerged as a key policy issue for the current U.S. presidential administration in an effort to support caregiving needs among families (17). Childcare needs require additional scrutiny, given that low income and minoritized populations disproportionately struggle with access to childcare. Furthermore, childcare needs are a key step in advancing gender equity (18–22). Given increasing attention to lack of diversity in medical research (23) and need for increased investment in women’s health research (22, 24), access to childcare warrants further study as a SDOH given its disproportionate relevance to women.

While childcare has been examined in the context of *pediatric* outcomes like early childhood development and asthma (25, 26), the impact of childcare needs on the health of *parents and caregivers* requires additional investigation. Prior work in the policy literature has established the relevance of childcare in parental *workforce* engagement (18, 27), but the impact of childcare on parent and caregiver *healthcare* engagement and health outcomes remains less clear. This scoping review aims to identify and summarize current knowledge on unmet childcare needs as a barrier to healthcare access for caregivers as well as to identify gaps in researching and addressing this SDOH.

## 2 Methods

### 2.1 Research question

The literature search was guided by the following research questions: What does the existing literature say about childcare needs as a barrier to healthcare access for caregivers of children? What are the gaps in understanding and addressing childcare needs as a social determinant of health for caregivers?

### 2.2 Search strategy

A literature search was conducted between June 2023 and August 2023. The search was limited to full journal articles published in the English language between January 1, 2000 and June 1, 2023. This time period was selected to encompass literature published since the Centers for Disease Control released Healthy People 2010 (28), which included the stated objective of eliminating health disparities and included access to healthcare for the first time as a leading health indicator. The search strategy included the following terms: social determinant of health, healthcare access, childcare, barrier, and disparity. The Boolean term “AND” was employed to specify terms essential to the potential literature review results (e.g., healthcare AND access AND childcare), and the Boolean term “OR” was used to maximize results for terms that may be used synonymously (e.g., healthcare OR “health care,” childcare OR “child care,” barrier OR disparity OR “social determinant of health”). The search query used for PubMed was, “(((((barrier) OR (disparity)) OR (“social determinant of health”)) AND (((healthcare) OR (“health care”)) AND (access))) AND ((childcare) OR (“child care”))) NOT (Review[Publication Type]),” and the search query used for Scopus was, “(“social determinant of health”) AND (access) AND (healthcare OR “health care”) AND (childcare OR “child care”) AND (LIMIT-TO (SRCTYPE, “j”)) AND (LIMIT-TO (SUBJAREA, “SOCI”) OR LIMIT-TO (SUBJAREA, “PSYC”) OR LIMIT-TO (SUBJAREA, “NURS”) OR LIMIT-TO (SUBJAREA, “MULT”) OR LIMIT-TO (SUBJAREA, “HEAL”) OR EXCLUDE (SUBJAREA, “MEDI”)) AND (EXCLUDE (DOCTYPE, “re”)) AND (LIMIT-TO (LANGUAGE, “English”)).” These search queries were tailored further for individual databases based on database capability, as some healthcare societies and health policy sites did not recognize advanced search operators or heeded zero results with long search queries. Customizing search queries by database permitted an adequate number of results per site searched.

### 2.3 Data sources

The primary search was conducted using PubMed to encompass the medical literature related to the study purpose. A second search was conducted with Scopus to expand the search across the following subjects: social sciences, psychology, nursing, multidisciplinary, health professions. Lastly, searches were conducted across professional healthcare societies and health policy fora to explore relevant medical research, position papers, and health policy briefs that may not have been included in the PubMed results. These websites included JAMA Health Forum (to include publications prior to PubMed indexing), the American College of Obstetricians and Gynecologists, the American College of Physicians, the American Academy of Family Physicians, the Society of General Internal Medicine, the Kaiser Family Foundation, the Commonwealth Foundation, and the Robert Wood Johnson Foundation.

### 2.4 Data extraction

Two independent reviewers downloaded search results from PubMed and Scopus into a spreadsheet. Results from other websites were manually transferred to a spreadsheet.

### 2.5 Data screening

**Figure 1:**
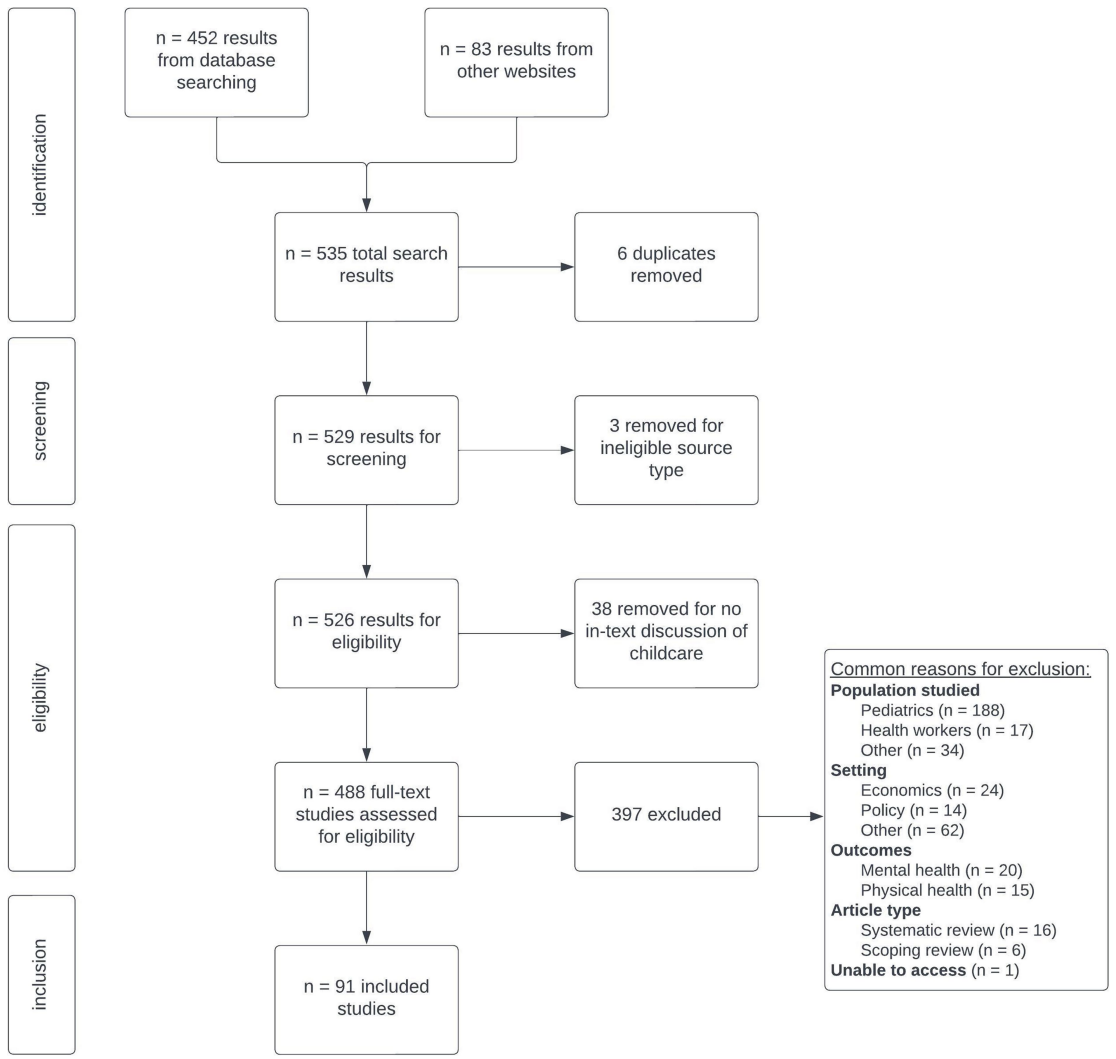
PRISMA flow diagram of studies reviewed for scoping review.

The search yielded 535 results, which were subsequently screened for duplicates, publication date, eligible source types, and presence of discussing childcare in full body-text (Figure 1). Six duplicate results were excluded from review. Three results were excluded as ineligible media, including 1 audio book and 2 reference sheets. Thirteen results were excluded after screening revealed that “childcare” was mentioned in relation to “maternal and child care” within institutional titles, or academic affiliation (e.g., a manuscript published by an author group from the National Maternal and Child Health Center in Phnom Penh, a large maternal and child health system, however article content was not about childcare) (29). Four results were excluded because childcare was solely mentioned in the abstract, not in the manuscript text itself. The 21 results that failed to mention childcare in their entirety came from databases that did not comply with Boolean expressions and were excluded during data screening. After this initial data screening, 488 results remained eligible for inclusion.

### 2.6 Data analysis

Two independent reviewers (MM and PT) reviewed the 488 eligible full-text studies for exclusion criteria. A third reviewer (AG) was consulted if the first two reviewers were not in agreement in their decisions for inclusion or exclusion. Studies were analyzed for eligibility based on population, setting, measures, and article type.

Studies were excluded based on population (*n* = 239) if the study population was not comprised of caregivers of children. For example, studies examining the role of childcare on health outcomes among pediatric subjects (children as recipients of childcare, *n* = 188) were excluded. Several studies explored the economic and/or policy impacts of childcare needs, namely impact of childcare on workforce engagement and cost of living; these studies were excluded as primary outcomes related to childcare were not healthcare-related (*n* = 100). Studies that examined the role of childcare on health outcomes outside of access to healthcare, such as the impact of childcare burdens on mental health or time for self-care outside of healthcare settings, were excluded to focus the analysis on the role of childcare as a logistical barrier to healthcare access and engagement (*n* = 35). Review papers were excluded (*n* = 22). One article was inaccessible to the reviewers.

After the eligibility review, 91 studies met criteria for analysis. Analysis followed best practices delineated by a scoping review methodology consensus group (30). Included studies underwent data extraction for the following characteristics: author, journal, year of publication, study country, clinical setting, study design, study population size, age range of study population, percentage of female participants, age range of children requiring childcare, and other SDOH or HRSN explored alongside childcare needs. A standardized Excel data charting form was utilized for data extraction of included studies. Basic qualitative content analysis utilizing an inductive approach was conducted to map salient themes from included studies. Two authors (MM and PT) conducted in-depth review of included studies to map to emerging themes. Analysis was iteratively reviewed among MM, PT, and AG to code themes and refine mapping. Two themes, theme #1 and theme #2, were defined *a priori* by the study team based on existing knowledge of the literature. Theme #3 was defined among studies initially categorized under theme #1, and theme #4 was defined among studies initially categorized under theme #3. Theme #5 was defined as a cross-cutting theme that emerged across the first four themes, as such studies categorized under theme #5 were double-coded in addition to original mapping along themes #1–4.

## 3 Results

**Figure 2:**
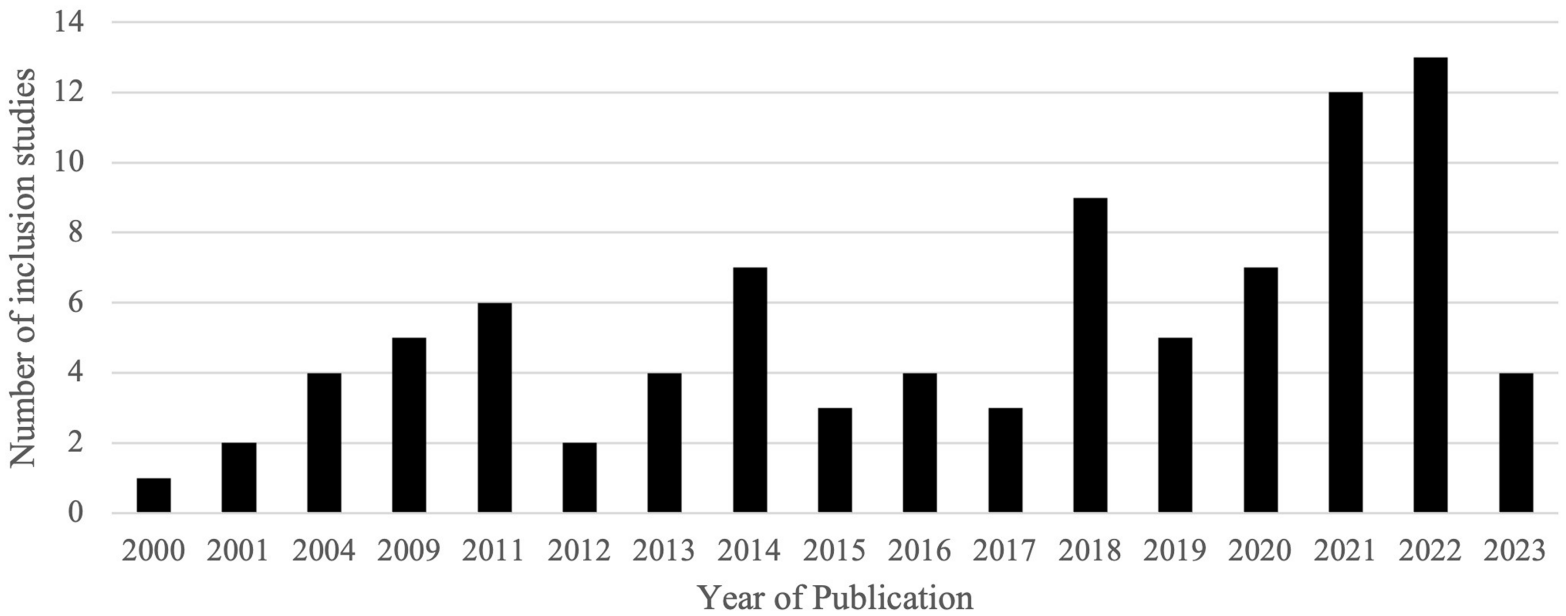
Inclusion study count by: publication year.

Characteristics of inclusion studies are listed in Table 1. Publications identifying childcare needs as a barrier to healthcare were noted to increase over time, as shown in Figure 2, with a lower volume of publications in 2023 due to the time frame of the literature search.

**Table 1:** Characteristics of all inclusion studies.

References	Year of publication	Country	Health topic	Number of participant	Age range of caregivers	% of female participant	Range of s number of children	Data collection	Study design	Discussion of childcare as a
Abdullahi et al. (55)	2009	UK	Cancer Screening	50	25–64	100	1–8	Focus groups and key informant interviews	Qualitative	Grouped
Ahmed et al. (10)	2001	USA	Access to Care	413	16–64	72	1–9	Surveys	Descriptive analysis of survey data	Alone
Aiken et al. (53)	2018	UK	Abortion Care	519	20–45	100	1–4	Retrospective review of resource website data	Mixed-methods	Grouped
Al-Azri et al. (60)	2022	Oman	Cancer Treatment	17	27–56	100	NA	Key informant interviews	Qualitative	Grouped
Alvarez et al. (66)	2022	USA	Access to Care	175	23–40	97	NA	Electronic medical record and dashboard	Quantitative program evaluation	Alone
Andrejek et al. (61)	2021	USA & Canada	Mental Health	23	20–40	100	1–2	Focus groups and key informant interviews	Qualitative	Alone
Appel et al. (68)	2004	USA	Substance Use Treatment	144	20–50	34	NA	Key informant interviews	Qualitative	Grouped
Arriens et al. (117)	2020	USA	Dermatology	23	21–72	87	NA	Focus Groups	Qualitative	Alone
Augusto et al. (56)	2013	Brazil	Cancer Screening	351	17–79	100	1–5	Surveys	Descriptive analysis of survey data	Grouped
Ayres et al. (37)	2019	Australia	Obstetric Care	218	18–40	100	NA	Surveys	Descriptive analysis of survey data	Alone
Barkin et al. (110)	2014	USA	Obstetric Care	31	25–35	100	1–3	Focus Groups	Qualitative	Alone
Bazzi et al. (77)	2016	Mexico	Substance Use Treatment	428	26–43	50	NA	Surveys and key informant interviews	Mixed-methods	Grouped
Beavis et al. (64)	2020	USA	Cancer Treatment	752	43–65	100	NA	Screening survey	Prospective cohort study	Alone
Benson et al. (76)	2020	USA	Cancer Screening and Treatment	204	NA	NA	NA	Surveys	Descriptive analysis of survey data	Grouped
Betancourt et al. (75)	2013	USA	Sexual Health and Family Planning	151	22–40	100	1–4	Focus groups and surveys	Mixed-methods	Grouped
Boehme et al. (45)	2014	USA	HIV and Obstetric Care	18	18–35	100	1–4	Surveys, key informant interviews, and focus groups	Mixed-methods	Grouped
Boom et al. (6)	2019	USA	Cancer Screening	69,139	21–64	100	NA	Secondary analysis of census data, surveys, key informant interviews	Mixed-methods	Grouped
Borland et al. (74)	2013	Canada	Substance Use Treatment	60	15–49	100	NA	Key informant interviews	Qualitative	Grouped
Bruno et al. (99)	2023	USA	Obstetric Care	165	18–45	100	1–3	Surveys, chart review, key informant interview	Mixed-methods prospective cohort study	Alone
Bryant et al. (138)	2009	Canada	Primary Care	2,536	18–54	61	NA	Surveys	Descriptive analysis of survey data	Grouped
Callister et al. (61)	2011	USA	Mental Health	96	17–39	100	2–6	Key informant interviews	Qualitative	Grouped
Canty et al. (62)	2019	USA	Mental Health	17	18–39	100	NA	Key informant interviews	Qualitative	Grouped
Chan et al. (44)	2021	Canada	Obstetric Care	218	20–35	100	1–3	Surveys and retrospective chart review	Cross-sectional analysis	Grouped
Chatterjee et al. (90)	2018	USA	Substance Use Treatment	14	24–51	79	1–5	Key informant interviews	Qualitative	Grouped
Clark et al. (54)	2011	USA	Cancer Screening	732	18–75	100	NA	Surveys and retrospective chart review	Program evaluation	Alone
Damle et al. (89)	2022	USA	Mistrust	21	20–49	100	1–4	Key informant interviews	Qualitative	Alone
Delvaux et al. (34)	2001	Austria, Denmark, Germany, Greece, Hungary, Ireland, Portugal, Spain, Sweden	Obstetric Care	2,494	20–35	100	1–4	Surveys and retrospective chart reviews	Case–control study	Alone
Fair et al. (40)	2020	UK	Obstetric Care	88	NA	100	NA	Surveys, key informant interviews, and focus groups	Mixed-methods	Grouped
Figueroa et al. (115)	2021	USA	Access to Care	7,514	19–64	55	NA	Surveys	Descriptive analysis of survey data	Alone
Fischer et al. (107)	2017	USA	Cancer Treatment	223	45–75	56	NA	Focus groups, prospective clinical data collection	Randomized controlled trial	Grouped
Fitzpatrick et al. (69)	2011	Canada	Cancer Treatment	33	18–75	73	NA	Key informant interviews	Qualitative	Grouped
FPM (65) (Policy Brief)	2018	USA	Primary Care	NA	NA	NA	NA	NA	Toolkit	Alone
Freedman et al. (59)	2017	USA	Cancer Treatment	18	36–87	100	NA	Key informant interviews	Qualitative	Grouped
Goodman (39)	2009	USA	Obstetric Care	509	18–45	100	NA	Surveys	Descriptive analysis of survey data	Alone
Handler et al. (70)	2018	USA	Access to Care	155	18–35	100	1–3	Key informant interviews	Qualitative	Grouped
Hanney et al. (71)	2022	USA	Physical Therapy	166	18–83	58	NA	Surveys	Cross-sectional analysis	Alone
Heaman et al. (31) (BMC Pregnancy and Childbirth)	2014	Canada	Obstetric Care	608	19–33	100	1–5	Key informant interviews and surveys	Mixed methods case–control study	Alone
Heaman et al. (32) (BMC Pregnancy and Childbirth)	2015	Canada	Obstetric Care	24	NA	92	NA	Key informant interviews	Qualitative	Grouped
Heaman et al. (33) (SAGE)	2015	Canada	Obstetric Care	26	15–37	100	NA	Key informant interviews	Qualitative	Grouped
Hildebrand et al. (108)	2018	USA	Diabetes	25	31–66	76	NA	Key informant interviews	Qualitative	Grouped
Hilton and Turan (72)	2014	Canada	Mental Health	199	NA	NA	NA	Surveys	Descriptive analysis of survey data	Alone
Hoskote et al. (73)	2022	USA	Access to Care	491	25–38	94	1–4	Surveys	Descriptive analysis of survey data	Alone
Huang et al. (88)	2023	USA	Diabetes	885	40–75	52	NA	Surveys	Descriptive analysis of survey data	Alone
Hulme et al. (58)	2016	Canada	Cancer Screening	37	28–69	100	NA	Focus groups and key informant interviews	Qualitative	Grouped
Inci et al. (105)	2020	Germany	Family Planning	307	18–63	100	1–10	Surveys	Descriptive analysis of survey data	Alone
Johnson et al. (87)	2021	USA	Surgery	135	36–65	71	NA	Surveys, retrospective chart review, key informant interviews	Mixed-methods	Alone
Jones et al. (101)	2021	USA	Substance Use Treatment and Obstetric Care	NA	NA	100	NA	NA	Commentary	Grouped
Kubo et al. (93)	2021	USA	Obstetric Care	27	19–39	100	1–3	Prospective chart review, key informant interviews	Mixed-methods program evaluation	Grouped
Kadaluru et al. (86)	2012	India	Oral Care	246	18–55	65	NA	Surveys	Descriptive analysis of survey data	Alone
KFF (Policy Brief) (63)	2004	USA	Access to Care	4,000	18–64	100	NA	Surveys	Descriptive analysis of survey data	Alone
KFFs (Policy Brief) (137)	2004	USA	Access to Care	4,000	18–64	100	NA	Surveys	Secondary cross-sectional analysis of survey data	Alone
King et al. (85)	2021	USA	Substance Use Treatment	16	NA	100	NA	Key informant interviews	Qualitative	Alone
Klaman et al. (51)	2019	USA	Family Planning and Substance Use Treatment	5,000	18–49	100	1–2	Surveys	Descriptive analysis of survey data	Alone
Lee et al. (84)	2013	New Zealand	Access to Care	NA	18–45	100	NA	Key informant interviews	Qualitative	Alone
Logan et al. (57)	2011	UK	Cancer Screening	48	18–65	100	NA	Key informant interviews	Qualitative	Grouped
Madden et al. (139)	2018	Australia	Hepatitis C	24	28–64	38	NA	Key informant interviews	Qualitative	Grouped
Maisa et al. (43)	2018	UK	Obstetric Care	16	18–44	100	1–3	Focus groups and key informant interviews	Qualitative	Grouped
Marshall et al. (83)	2021	USA	Access to Care	208	NA	100	NA	Focus groups, key informant interviews, observation of healthcare delivery	Qualitative and Implementation Evaluation	Alone
Martis et al. (41)	2018	New Zealand	Obstetric Care and Diabetes	60	27–38	100	NA	Key informant interviews	Qualitative	Alone
Minian et al. (52)	2016	Canada	Substance Use Treatment	23	50–59	100	NA	Focus groups	Qualitative	Alone
Moreau et al. (102)	2018	USA	Mental Health	40	NA	85	NA	Key informant interviews	Qualitative	Grouped
Morgan et al. (98)	2022	UK	Obstetric Care	164	27–39	100	1–2	Surveys	Descriptive analysis of survey data	Alone
Morrow et al. (92)	2004	USA	Substance Use Treatment	123	24–70	100	NA	Focus groups, key informant interviews, and surveys	Mixed-methods descriptive analysis	Alone
Nicklas et al. (106)	2011	USA	Diabetes	25	30–45	100	1–4	Focus groups and key informant interviews	Qualitative	Alone
Nock et al. (82)	2023	USA	Dermatology	16,986	38–67	74	NA	Surveys	Descriptive analysis of survey data	Alone
Nothnagle et al. (36)	2000	USA	Obstetric Care and Access to Care	6,364	20–35	100	1–5	Key informant interviews	Qualitative	Grouped
Nyamathi et al. (50)	2011	India	HIV	39	20–45	100	1–4	Focus groups	Qualitative	Alone
Pandey et al. (95)	2022	Canada	Access to Care	37	29–48	76	1–4	Focus groups	Qualitative	Grouped
Pearson et al. (94)	2012	Honduras	Access to Care	220	30–50	78	NA	Surveys	Descriptive analysis of survey data	Alone
Peahl et al. (97)	2021	USA	Obstetric Care	253	25–37	100	1–3	Retrospective chart review and surveys	Program evaluation	Grouped
Peahl et al. (35)	2022	USA	Obstetric Care	19	23–35	100	1–3	Key informant interviews	Qualitative	Alone
Penaranda et al. (104)	2014	USA	Cancer Screening	21	37–65	100	NA	Focus groups	Qualitative	Alone
Ranji et al. (16)	2017	USA	Access to Care	2,751	18–64	100	NA	Surveys	Descriptive analysis of survey data	Alone
Rebbeck et al. (112)	2022	USA	Genetics	NA	NA	NA	NA	Conceptual Framework	Theories, Models, and Frameworks	Grouped
Rivers et al. (42)	2020	USA	Diabetes and Obstetric Care	39	NA	100	NA	Focus groups	Qualitative	Alone
Robiner et al. (111)	2009	USA & Canada	Diabetes	285	20–40	53	NA	Surveys	Randomized controlled trial	Alone
Rodin et al. (81)	2019	USA	Access to Care	2025	NA	100	NA	Focus groups, key informant interviews, and surveys	Mixed-methods	Grouped
Rosenberg et al. (103)	2022	USA	Obstetric Care	68	NA	93	1–5	Surveys, key informant interviews	Mixed-methods	Alone
Sakai-Bizmark et al. (47)	2022	USA	Obstetric Care	1,109,785	23–35	100	NA	State database	Cross-sectional analysis	Grouped
Schwartz et al. (38)	2021	Canada	Obstetric Care	57	27–37	100	1–3	Surveys	Descriptive analysis of survey data	Alone
Shippee et al. (96)	2014	USA	Emergency Medicine	4,626	30–45	66	NA	Survey and administrative data	Cross-sectional analysis	Grouped
Sinha et al. (49)	2022	USA	Diabetes	36	26–35	100	2–4	Focus groups, key informant interviews	Qualitative	Alone
Slaunwhite (80)	2015	Canada	Mental Health	4,134	30–40	65	NA	Surveys	Cross-sectional analysis	Grouped
Stirling et al. (79)	2021	Canada	Access to Care	8	NA	100	1–8	Key informant interviews	Qualitative	Alone
Stirling et al. (91)	2022	Canada	Access to Care	11	NA	100	1–8	Key informant interviews	Qualitative	Alone
Van Ryswyk et al. (48)	2016	Australia	Diabetes	207	27–38	100	NA	Surveys	Randomized controlled trial	Alone
Webb et al. (46)	2014	USA	Obstetric Care	471	19–32	100	NA	Surveys	Descriptive analysis of survey data	Alone
Weith et al. (100)	2023	USA	Mental Health	85	NA	NA	NA	Surveys	Descriptive analysis of survey data	Alone
Welch et al. (109)	2009	Australia	Obesity Medicine	59	18–45	100	NA	Focus groups and key informant interviews	Qualitative	Alone
White-Means et al. (78)	2020	USA	Cancer Treatment	5	54–68	100	NA	Focus groups	Qualitative	Grouped
Wong et al. (114)	2021	USA	Access to Care	NA	NA	NA	NA	Conceptual Framework	Theories, Models, and Frameworks	Grouped

Inclusion studies were primarily published in the United States, but several more countries were represented, including Canada, the United Kingdom, and Australia, among others. Caregiver ages typically ranged from about 20–50 years, as listed in Table 1, and most study populations were comprised of majority female participants. Inclusion studies represented a wide array of clinical contexts, including primary care and specialty care, outpatient and hospital medicine, research settings, and community outreach. Studies included many data sources, encompassing focus groups (*n* = 22), key informant interviews (*n* = 42), surveys (*n* = 43), chart reviews (*n* = 7), resource website or census data (*n* = 2), observation of healthcare delivery (including electronic medical record dashboard, *n* = 4), and conceptual framework (*n* = 2). Study design was similarly heterogeneous. More than half (*n* = 50) included some component of qualitative analysis. Among 48 studies that included quantitative analysis, 50% (*n* = 24) were descriptive analyses of survey data and 11 utilized other observational designs. Only 3 inclusion studies were randomized controlled trials, and notably these three studies studied childcare needs alongside trial engagement, not as a study exposure or outcome.

### 3.1 Theme #1: Childcare needs are a barrier to attending healthcare appointments

The majority of studies included (*n* = 71) examined childcare as a logistical barrier to attending health appointments among caregivers (Table 2). Childcare needs were identified across a wide distribution of health topics and clinical settings, including obstetric care (*n* = 17), access to care (*n* = 16), cancer screening (*n* = 7) and cancer treatment (*n* = 6), diabetes (*n* = 5), mental health (*n* = 5), family planning (*n* = 2), HIV care (*n* = 2), and more, as described in Table 2.

**Table 2:** Illustrative quotes from Theme #1: childcare needs are a barrier to attending healthcare appointments.

Health topic	Illustrative quotes
Obstetric care (*n* = 17)	“Regardless of referral and engagement status, the factors identified as influencing participant engagement were time restraints, lack of childcare support, and encouragement by family and health care professionals.” (36).
	“Providing free services, in places with good transport links, on-site childcare, high visibility through good marketing and de-centralized services to reduce the distances women have to travel were all considered vital to improve service accessibility.” (39).
	“Financial and logistical barriers, such as lack of time, transportation, or child care, have been identified among women’s clinic patients.” (38).
	“While lack of transportation and child care are widely recognized as two important barriers to obtaining prenatal care for low-income women in general, this study demonstrated that “having transportation problems” and “having child care problems” were distinguishing factors between women who obtained adequate prenatal care and those who did not.” (30).
	“If you have to take your stroller and your three kids and take a bus exchange … the benefit is not worth the problems that it creates.” (Patient quote) (31).
	“‘For me, because of the fact that I have other kids, finding buses that are easy access to get a stroller onto [is a problem] … And trying to plan what it is that we are going to do in the doctor’s office when we are waiting for an hour and a half with my kids.’ (G4P3, 27 years).” (Patient quote) (32).
	“Lack of time, responsibility of organizing appointments, time off work and difficulty accessing childcare were among barriers participants mentioned.” (42).
	“Patients and HCWs identified structural issues with prenatal care delivery. Decentralized resources made fulfilling medical and nonmedical needs challenging. In addition, many patients reported difficulty balancing other obligations, such as work and childcare, with prenatal services.” (34).
	“Obstacles to utilizing postpartum healthcare for homeless mothers, beyond securing food or housing, include distance to care coupled with lack of transportation, irregular insurance coverage, distrust due to poor treatment by providers, lack of childcare, low health literacy, and fear of being reported to Child and Family Services for homelessness or substance abuse.” (46).
	“Lack of time and lack of childcare were barriers to seeking help for 38 and 23% of the participants, respectively.” (37).
Access to care (*n* = 16)	“Collaboration with community-based organizations to address lack of childcare creates a way for patients to access medical care instead of foregoing care, and a no-patient cost campus childcare center was used by patients when made available.” (65).
	“‘Competing priorities get in the way of my ability to be healthy. Between work, childcare, and home duties—there is little time left over for me to take care of myself. I worry about making sure that everyone else is well taken care of that I often leave myself out.’ (Decatur, GA).” (Patient quote) (69).
	“In addition to cost, low-income women are more likely than higher-income women to report that lack of insurance, transportation problems, and lack of childcare also contribute to delays in obtaining care.” (137).
	“Latinas (15%) are also more likely to report that problems in obtaining childcare resulted in delayed or unmet care.” (62).
	“A lack of childcare was raised as a barrier to accessing health care by most participants. Sole mothers can find health emergencies or hospitalization extremely difficult to deal with if they had no ready help with childcare: ‘Yeah, but I was in really bad pain, but there was no way I could ring an ambulance because I had my three-year-old son, you know. So, I had to drive all the way to [locality] in this mega-pain, track down my friend and then be driven to the hospital.’ (#2)” (83).
	“‘If we are looking at barriers of care for women, childcare is a real issue. We’re really fortunate that the [non-VHA facility] runs a free childcare service…. We went over, asked them if they would extend that to veterans … So now there’s up to 2 h of free childcare if you have an appointment … That was one thing that we did to try to increase access.’ - Women’s Health Interview, Urban Site A.” (Leader/Administrator interview) (82).
	“Despite expansions in coverage, many women still experience cost-related barriers to care and face medical bills that force them to make difficult tradeoffs. For some women, logistical problems such as lack of childcare or difficulty taking time off work pose barriers of care.” (16).
	“Because of a general lack of affordable child care, many Strong Start participants had no alternatives to bringing children to visits, resulting in skipped visits, especially when children were not actively welcomed.” (80).
	“Some women reported missing healthcare appointments because they did not have access to childcare. One participant declined visiting a psychiatrist, because of limited childcare: ‘I seriously considered it, but my kids were little, and I had no one to take care of them.’ (Participant 5).” (90).
Substance use treatment (*n* = 8)	“Given the reality of childcare and financial constraints, couples often pooled resources so one partner could access [residential drug] treatment alone while the other looked after children and worked to meet financial obligations.” (76).
	“In clinical practice, barriers specific to treatment of opioid use disorder (OUD) for these patients include the need for childcare during visits, concerns about accidental ingestion of prescribed opioid agonist medications by children, and the distance between family shelters and treatment sites.” (89).
	“Participants described how pregnant patients often had other children to care for, making attending daily dosing appointments especially difficult as no participating clinics offered childcare services.” (84).
	“An additional suggestion made by some clients to improve accessibility of the [smoking cessation] clinic for women was to offer childcare services. ‘For women especially… [it would be helpful] if it would be more accessible after hours and daycare would be HIGHLY appreciated.’ FG1.” (51).
Cancer screening (*n* = 7)	“Practical difficulties such as inconvenient appointment times and lack of childcare were perceived as a barrier to attending screening, especially for single mothers with young children.” (54).
	“…Another frequently cited barrier [to cervical cancer screening] involved the time constraints imposed by job responsibilities and childcare.” (55).
	“Women were more likely to report a recent Pap smear at study entry if they were younger (aged 18–49), had social support for childcare, had access to transportation, and lacked housing concerns, including facing eviction.” (53).
	“Competing priorities with work and childcare were of greatest concern for young Chinese mothers, whereas transportation, language, and cultural barriers were the challenges voiced by older unilingual Chinese women.” (57).
	“Various barriers were identified by some women for non- attendance for cervical screening. The main barriers were attributed to practical factors such as the timing of the appointments, issues of time and having to find child care.” (56).
Cancer treatment (*n* = 6)	“‘I had no time to look after myself nor my health, and no one to help me. My elder daughters are attending colleges and universities and thus busy studying; besides, I have other young children to take care of and house chores to do.’” (Patient quote) (59).
	“Another woman stated, ‘I have to pay for child care and I live too far away, but suggests a once-a-week child care program to enable women to bring their children if necessary.’” (68).
	“In addition to chemotherapy toxicity requiring dose interruption or modification, a substantial proportion of women (≈39%) reported logistical (transportation, childcare) or personal preference barriers that limited their compliance.” (58).
Diabetes (*n* = 5)	“In multivariable logistic regression models adjusting for age, gender, insurance status, education, and income, Hispanic individuals (OR = 6.57; 95% CI, 1.67–27.8) were still more likely than non-Hispanic White individuals to report delaying getting medical care due to not being able to get child care.” (87).
	“Provision of a free health shuttle for clinic appointments, a hospital crèche for child care, an efficient appointment system reducing waiting times and partner and extended family welcomed at teaching sessions and clinic appointments contributed to women’s ability to perform CBG (capillary blood glucose) testing.” (40).
	“‘I had a 1-year-old and a newborn, it’s hard to find childcare if you want to go to the doctor because some Dr’s offices do not allow you to bring children so that was a huge issue too. (Participant 5, unscreened, delivered ≥1 year).’” (Patient quote) (48).
	“The most frequently indicated barrier was not having enough time (*n* = 24/33, 73%), followed by inadequate or non-availability of childcare (*n* = 10/33, 30%), and a need to focus on the health of the baby (*n* = 10/33, 30%).” (47).
	“There were several barriers mentioned, but the vast majority mentioned the following: 0.2. The need for childcare (68%): ‘Childcare will be a problem for me because I have a baby and an 8-year-old, so it would be hard to pay attention to the session while I’m nursing or changing a diaper.’” (41).
Mental health (*n* = 5)	“Future perinatal patients may be reluctant to attend in-person BA [Behavioral Activation] sessions because they do not have childcare: ‘This barrier is not COVID-related; it’s just always been a barrier that you cannot find childcare.’ (Stakeholder_12_Canada).” (66).
	“This pattern of responses suggests that [mental health] services are readily available and accessible for parents; however, child care was the parenting service least often endorsed, which likely limits the ability of parents with young children to access the programs that are offered.” (71).
	“Although statistically insignificant, women were 51.1% more likely to report that a lack of transportation or childcare limited their ability to obtain mental health services. Women were also 17.6% more likely then men to report that personal-family responsibilities prevented them from obtaining help for their mental health issue.” (79).
Family planning (*n* = 2)	“81% of medical and program directors perceive the lack of facility equipment and supplies (eg, obstetric, gynecological, immunization), and childcare for the children of female patients to be the most significant barriers to onsite integration of [reproductive and sexual health] services.” (50).
HIV (*n* = 2)	“Our focus group analysis from the perceptions of rural women living with AIDS reveal that there are several barriers to antiretroviral therapy adherence including illness-related factors, financial constraints, problems with traveling long distances to receive therapy, childcare issues, stigma, and psychological problems.” (49).
	“The majority of participants described transportation as impacting HIV care utilization, putting a significant burden on their finances, and competing with childcare costs.” (44).
Primary care (*n* = 2)	“In addition to investing more resources in the healthcare system to ensure sufficient resources are available to provide needed healthcare, public policy solutions such as providing child care and other social supports will help reduce unmet healthcare need.” (138).
Abortion care (*n* = 1)	“‘I am only 2 weeks pregnant, I already have 3 kids and I am a single working mum. I am unable to go to the hospital as I do not have the funds to pay for childcare while I would be in there. I am unable to take time off work and I cannot tell my family so there is no one I can ask to look after the kids. I really need to do this in my own home.’” (Patient quote) (52).
Dermatology (*n* = 1)	“With regards to structural barriers beyond cost, Black patients with CISDs were significantly more likely than White patients to delay medical care because of transportation issues (aOR, 3.27; 95% CI, 2.72–3.92), not being able to take time off work (aOR, 1.23; 95% CI, 1.02–1.49), needing to provide childcare (aOR, 1.67; 95% CI, 1.18–2.31).” (81).
Emergency medicine (*n* = 1)	“Rather than being associated with ED use simply as a correlate or expression of financial vulnerability, or indirectly as associated with poorer mental health, trauma, or broader utilization patterns, such non-financial barriers indicate practical difficulties that are embedded in the tangible constraints embedded in individuals’ everyday lives (e.g., child care, transportation, office hours, and others).” (95).
Hepatitis C (*n* = 1)	“Participants spoke about the difficulty of getting ancillary support such as help with transport or childcare services that would allow them to attend [Hepatitis C Virus] treatment services, and they emphasized the frustration they experienced in their attempts to co-ordinate their care.” (117).
Oral care (*n* = 1)	“Among the female subjects, 19.5% (48), said that they had not visited the dentist because it was difficult to take time off from child care duties.” (85).
Physical therapy (*n* = 1)	“Other factors that may influence an individual’s ability to participate in [physical therapy] care include childcare during treatment (33.7%), work schedule, (42.2%), and caring for other family members (33.1%).” (70).
Sexual health (*n* = 1)	“The highest survey-rated barriers [to sexual and reproductive health services] were: (1) inability to pay for services, (2) providers not speaking Spanish, (3) difficulty obtaining child care, and (4) fear of receiving poor-quality services (see Table 5).” (74).
Surgery (*n* = 1)	“This study aimed to characterize patient-reported concerns about undergoing surgical procedures during the pandemic… Compared with white participants, nonwhite participants had nearly 5 times the odds of having concerns about childcare or other dependent care (*p* = 0.01) and 2.5 times the odds of having concerns about transportation (*p* = 0.04); there was no significant difference in concern about finances (*p* = 0.09).” (86).

Across these studies, 52 specifically examined childcare barriers among women. Specific clinical contexts included obstetric care (*n* = 17) settings including access to (31–34) and experiences with prenatal care (35, 36), perinatal mental health (37–39), perinatal healthy weight services (40), management of gestational diabetes (41, 42), access to vaccinations during pregnancy (43), postpartum cardiovascular risk follow up (44), adherence to HIV care (45), and the prevention of preterm births (46). One study identified lack of childcare as a contributor to decreased rates of postpartum hospital readmissions in homeless women (47). Other clinical contexts where childcare needs posed a barrier to women’s healthcare access included prevention of diabetes after pregnancies complicated by gestational diabetes (48, 49), access to HIV/AIDS care (50), access to sexual and reproductive healthcare for women enrolled in opioid use disorder treatment programs (51), access to smoking cessation programs (52), and access to abortion services (53). Childcare needs were also identified as a barrier to accessing breast and cervical cancer screening and treatment (6, 54–60).

A few studies also reported childcare as a logistical barrier to healthcare needs for the general caregiver population, not just for women. This barrier was identified in access to substance use treatment (*n* = 8), mental healthcare services (*n* = 5), dermatology visits (*n* = 1), elective surgeries (*n* = 1), oral care (*n* = 1) physical therapy (*n* = 1), and emergency department utilization (*n* = 1).

In theme #1, studies either considered unmet childcare needs as a discrete health-related social need (*n* = 37, 52.1%) or grouped childcare alongside other social needs (*n* = 34, 47.9%) in analysis and discussion. The 34 studies that examined childcare alongside other social needs often evaluated the affordability and accessibility of arranging childcare alongside transportation, income level, and language barriers. Among the 37 studies that discretely evaluated childcare as an individual barrier, 35 (94.6%) concluded that unmet childcare needs delayed care. Common reasons for missing or deferred appointments because of unmet childcare needs included limited affordability of childcare support (6, 10, 31–34, 36–40, 43–45, 48–52, 55–59, 61–89), distractions from children during care (42, 60, 84, 90–92), and limited flexibility of appointment times (52, 93–96). The remaining two studies did not find a significant association between childcare and delayed care (35) or noted uncertainty over childcare as a barrier to healthcare access (47).

Compared to studies that grouped childcare with other barriers, studies that examined childcare needs as an individual barrier to healthcare access were more likely to provide direct recommendations for interventions for childcare needs, including on-site childcare facilities at the point of healthcare access (31–33, 39, 51, 52, 55, 66, 67, 70, 72, 81, 83, 90, 92), subsidizing childcare (76), or dedicated case management for childcare services (50, 54). Four studies further examined how provision of childcare increased access to or engagement with healthcare services. One study found that patients receiving free onsite childcare were more likely to complete blood work for gestational diabetes (41); another demonstrated that a health center with onsite childcare facilitated medication adherence for women living with AIDS (50). Additionally, one study found that having social support for childcare permitted regular cervical cancer screening (54) and another identified free childcare as a potential intervention to increase access to risk reduction services for repeating a preterm birth (46).

### 3.2 Theme #2: Alternative care delivery models may alleviate barriers posed by childcare needs

Thirteen studies discussed the role of alternative healthcare delivery methods in contrast to traditional in-person visits onsite at healthcare facilities to circumvent logistical barriers to appointments, including childcare needs (Table 3). The most common alternative care delivery method reported was virtual care through telemedicine (*n* = 9). All but one of the studies focused on pregnant women seeking obstetric (*n* = 5) (35, 67, 97–99) or psychiatric care (*n* = 3) (93, 100, 101). While most studies (*n* = 8) concluded that virtual care allowed for greater patient engagement in women’s healthcare services by bypassing childcare barriers, one study discussed the mixed effectiveness of virtual care in overcoming childcare needs since children could distract from virtual care engagement even within the home during virtual visits (98). Though many of the studies included pregnant women who were predominantly white and from higher socioeconomic backgrounds (97–99), some studies further explored the benefits of virtual care in increasing access to mental health care for marginalized populations, including refugees (100), women receiving care in the Veteran Affairs Health System (102), and pregnant women with substance use disorders (101).

**Table 3:** Illustrative Quotes from Theme #2: alternative care delivery models may alleviate barriers posed by childcare needs.

Health topic	Illustrative quotes
Obstetric care (*n* = 7)	“Women appreciated the convenience of the intervention and the ability to engage without having to attend classes or arrange childcare. We observed significant improvements in pre-postintervention scores for depression symptoms, perceived stress, sleep disturbance, and mindfulness.” (92).
	“In comparison, 69 respondents also identified challenges to attending in-person visits, including taking time off work (50.7%), childcare concerns (24.6%), access to transportation (7.2%), and money for gas (10.1%), as well as concern about exposure to SARS COV-2 through interaction with the healthcare system (7%).” (96).
	“Participants envisioned a robust network of care navigators, such as community HCWs or doulas, who could help patients accomplish health care tasks and feel supported. They also identified flexible prenatal care models (eg, telemedicine, group care, and community-based clinics) and expanded hours, availability of childcare services, and colocation of medical and nonmedical services as avenues for reducing access barriers.” (34).
	“Related to patient- and family-centered convenience, caregivers discussed transportation, childcare, and scheduling as typical burdens and inconveniences of in-person office visits eased by the [mobile medical clinic]. One participant noted the convenience of not needing to coordinate travel or childcare for other children while home schooling during COVID-19: ‘Because I have two other kids that are toddlers and they do virtual school, so it’s hard to go all out once over there. And then also taking the baby out.’ – Respondent #11.” (102).
Mental health (*n* = 3)	“Because mental health care typically involves regular, repeated sessions over time, travel burden for consistent care is greater than for occasional or isolated care visits, such as an annual Pap. Stakeholders saw women veterans as being particularly poised to benefit from telemental health, owing to responsibilities associated with childcare, spousal care, and elder caregiving.” (101).
	“One provider shared their experience with both the drawbacks and benefits of telemental health regarding childcare: ‘For clients who have children, caring for them during the session has more options from home than in the office, though the distractions remain.’ Overall providers shared that on the one hand clients do not have to find childcare, which can be a barrier with in-person services, however, having children at home during sessions can increase distractions that were not present for in-person appointments.” (98).
Substance use treatment (*n* = 3)	“Participants staying in the family shelters, even those not receiving treatment through the [shelter-based opioid therapy (SBOT)] program, expressed an understanding of the value of SBOT. The most common reason for enrolling in SBOT or desiring to switch into SBOT was convenience, since all appointments were in the shelter and would not require transportation or childcare (because clinic staff could watch children during visits).” (89).
	“Opportunities for all patients with substance use disorders include virtual platforms presenting positive opportunities for treatment. They are time efficient, eliminate transportation barriers, and potentially reduce childcare barriers.” (100).
Cancer screening (*n* = 1)	“The benefits of self-sampling compared with the Pap test were ease, convenience, practicability, less embarrassment, and the lack of need for child care.” (103).

One study deployed a mobile medical clinic as a response to health access barriers in maternal–infant care during COVID-19 (103). By addressing proximity to care, the mobile clinic was shown to reduce childcare barriers for women receiving care, particularly among Black and Latina mothers. Another study illustrated how childcare needs were circumvented with self-screening for cervical cancer in a population of Hispanic women (104). The use of outpatient methadone maintenance therapy in lieu of traditional residential drug treatment programs was identified as one possibility to ease the burden of childcare on access to opioid use disorder (OUD) treatment (77). Furthermore, in an attempt to address the disproportionate burden of OUD among people experiencing homelessness, a study identified shelter-based opioid treatment as an alternative treatment modality that that eased the burden of childcare needs (90).

### 3.3 Theme #3: Childcare needs are a barrier to participating in medical research

Childcare as a barrier to healthcare access was noted to extend beyond clinical appointments to the ability to engage in medical research. Of the studies that investigated the effect of childcare in caregivers’ research participation (*n* = 11), 7 cited childcare as a barrier to research participation, and 4 directly addressed this barrier by providing childcare to facilitate enrollment of caregivers in research studies (Table 4). All four studies that used childcare resources as an incentive for research participation solely recruited women as the target population for research studies investigating obstetric outcomes and women’s health. Three studies provided on-site childcare to increase study participation, including a trial identifying risk factors for repeat preterm birth (46), interviews exploring barriers to diabetes screening among women with gestational diabetes (49), and family planning group visits among female refugees in Germany (105). One study provided compensation for childcare to supplement participation in focus groups discussing type 2 diabetes prevention for women with history of gestational diabetes (106).

**Table 4:** Illustrative quotes from Theme #3: childcare needs are a barrier to participating in medical research.

Health topic	Illustrative quotes
Diabetes (*n* = 4)	“The focus groups lasted 70 min on average and were digitally recorded and transcribed for analysis. Focus group participants received compensation for childcare and transportation costs.” (105).
	“[For study participation], participants received free parking or a voucher for public transit, childcare on site if desired, and $50 cash compensation for their time.” (48).
	“‘One has to work… I take care of my grandson… so my daughter can work. I do not get paid, but it is the only way to help each other.’” (Patient quote) (107).
Obstetric care (*n* = 2)	“Thirty-one of 33 women completed the study and therefore received a $50 gift card for groceries. Study completion entailed participating in a single, 2-h long focus group discussion. Two women dropped out because they were unable to secure child care at the time of their scheduled focus group discussions.” (109).
Cancer treatment (*n* = 1)	“Patient transportation and childcare are other significant barriers that need to be addressed. Patients often have difficulty finding transportation to the cancer center for treatment. They also care for children and grandchildren while their caregivers are working. Budgeting for patient transportation including Uber or Lyft vouchers and childcare assistance may support patient participation.” (106).
Dermatology (*n* = 1)	“Some also expressed the need to have study visits combined with their regular office visits. Advance notice to aid in making arrangements for childcare or work was also noted.” (116).
Family planning (*n* = 1)	“Each presentation included information on contraceptive options, breast cancer self-examination and maternal health within the German healthcare system. The events were for women only, in order to build trust within the group and provide privacy during the study questionnaire. Furthermore, in order to ensure that all women had the chance to participate, the management organized childcare for the children.” (104).
Genetics (*n* = 1)	“Subjective norms and motivation to comply: physical access may be limited by individual needs including childcare, eldercare, time off from work, transportation. Cultural perspectives and beliefs of family or friends and other support networks including culturally based concerns such as ethnic or tribal identity or individual genetic privacy.” (111).
Obesity medicine (*n* = 1)	“It became clear early on in the research process that those women we most wanted to recruit into our research were the hardest to access. These were the women who, for a number of reasons including childcare and employment commitments, may have been least likely to attend a focus group.” (108).

Studies that identified childcare as a barrier to research participation often sought to recruit racially diverse or socioeconomically disadvantaged individuals (*n* = 4). Two studies targeted recruitment of Latino participants (107, 108), one study recruited female refugees residing in Berlin (105), and one study aiming to recruit women from socioeconomically disadvantages areas concluded that, “…those women we most wanted to recruit into our research were the hardest to access” (109). Additionally, two studies specifically noted women as a vulnerable population for unmet childcare needs limiting research participation (110, 111). While most of studies examining childcare barriers to research were one-time focus group or interviews for participants (*n* = 5), one study assessed research participation barriers over a five-year period and concluded that barriers like childcare can longitudinally affect research engagement (111). A framework for promoting equitable inclusion in genetics and genomics research identified childcare as a need to be considered in expanding diverse research participation (112).

### 3.4 Theme #4: Childcare needs were exacerbated during the COVID-19 pandemic

Nine studies examined how the COVID-19 pandemic and associated childcare crisis (113) altered the way that caregivers access healthcare (Table 5). The effect of unmet childcare needs on healthcare access was made even more pronounced as closed schools and daycare centers forced parents and caregivers to isolate with their children and assume increased responsibilities for caregiving previously provided in institutional settings. Aside from immediately impacting changes in childcare, one inclusion paper explores the Public Health 3.0 framework, which consists of a collaborative effort across local public health institutions to identify and monitor health disparities and identifies childcare as a critical indicator for pandemic preparedness and response that requires further evaluation to determine the pandemic’s social impact on public health (114).

**Table 5:** Illustrative quotes from Theme #4: childcare needs were exacerbated during the COVID-19 pandemic.

Health topic	Illustrative quotes
Access to care (*n* = 4)	“The pandemic severely restricted access to childcare, and our study highlights unintended health-related consequences of the dissolution of the childcare infrastructure. Although school closures and stay-at-home orders were implemented to protect the public’s health, there were clearly significant consequences of these governmental actions on family health and welfare.” (72).
	“With the temporary closures of public schools, and daycare centers, families had limited options for childcare for their additional children. Participants also did not have extended family (e.g., mothers, sisters) in Canada and many women did not feel comfortable asking friends to watch their children, given their friends’ own responsibilities and the risk of spreading the virus.” (78).
Obstetric care (*n* = 2)	“There was often overlap with patient- and family-centered care and convenience, as the components of the [mobile medical clinic (MMC)] model which provided convenience for transportation, childcare, privacy, ease of scheduling and communication also allowed many caregivers physical space and reassurance around COVID-19 safety during the height of the pandemic in 2020: ‘Honestly, the [MMC] is just better, with no other patients on the [MMC]. So I just you know, I’m just scared because people start coughing, do not cover their mouth. At the clinic, that’s what I’m worried about. But the [MMC] is literally just you, so you do not have to worry about much.’ – Respondent #3.” (102).
Mental health (*n* = 1)	“Similarly, a provider stated that, ‘we had childcare at [the hospital]’ that parents could leave their kids at during psychotherapy sessions but that this was closed due to COVID-19 and explained that ‘I have no idea when that would be able to be opened up. I feel like it would be very long [in the future]. So, finding other childcare would be a big barrier.’ (SP_06_Canada).” (66).
Mistrust (*n* = 1)	“Some participants described canceling health care appointments because of new restrictions within health care facilities that prohibited children from accompanying their parents. Others described using home remedies to treat ailments (including symptoms of COVID) instead of seeking out health care services because of the difficulties of finding, and affording, childcare.” (88).
Substance use treatment (*n* = 1)	“Virtual platforms may not only promote patient engagement in treatment, but may also provide easier access to treatment by eliminating barriers associated with transportation and childcare, and serve as a means to ensure privacy for some patients.” (100).
Surgery (*n* = 1)	“Patients reported uncertainty and frustration regarding the delay of their care and future scheduling. This uncertainty made arranging childcare, travel, and planning for the financial impacts of elective surgery difficult. Possible unemployment was tied to fears of losing insurance coverage, and travel concerns were often related to concerns about dependent care.” (86).

Studies reported a significant association between childcare barriers and delayed healthcare for low-income parents (73, 115). New public health restrictions deepened unmet childcare needs as women, particularly Latinx immigrant women (89), struggled with healthcare access when they were unable to bring children to appointments or secure childcare. Lastly, the impact of the pandemic on unmet childcare needs persisted long after the return to full health systems operations; one study noted that when non-essential surgeries resumed post-pandemic, childcare continued to serve as a barrier to scheduling surgeries, and that nonwhite participants were five times as likely to have childcare concerns (87).

The exacerbation of childcare needs during COVID-19 also galvanized innovations in alternative methods of healthcare delivery, as explored in theme #2 above. The mobile medical clinic introduced in theme #2 was one response to the heightened childcare needs during COVID-19 that successfully helped to circumvent this burden by bringing care closer to patients in their communities (103). Virtual healthcare also grew exponentially during the COVID-19 pandemic, and pregnant and postpartum patients with substance use disorders were reported to benefit from transitioning to telehealth by reducing in-person barriers, including childcare (101). Conversely, telehealth was noted to be potentially insufficient to close the gap in existing healthcare disparities due to residual structural inequities like the digital divide (116); one study that recognized childcare as a reason for delaying care also noted low rates of telehealth use among low-income, Black, and Latinx patients (115).

### 3.5 Theme #5: Childcare needs disproportionately impact marginalized populations

Vulnerable populations grappling with unmet childcare needs across all themes included women (*n* = 66), racial/ethnic minorities (*n* = 28), and low-income families and individuals (*n* = 10). Studies also highlighted the relevance of childcare needs among immigrants and refugees, residents of geographically isolated areas, and people with substance use disorders (Table 6). Twenty-eight total studies explored the impact of childcare on non-white Hispanic patients (*n* = 11), Black/African-American patients (*n* = 12), and indigenous patients (*n* = 5), among which 12 studies applied an intersectional approach to understanding childcare among women of color.

**Table 6:** Illustrative quotes from Theme #5: childcare needs disproportionately impact marginalized populations.

Vulnerable population	Sub-group	Illustrative quote
Racial & ethnic minorities (*n* = 28)	Non-White Hispanic (*n* = 11)	“Latinas (15%) are also more likely to report that problems in obtaining childcare resulted in delayed or unmet care.” (62).
	Black/African-American (*n* = 12)	“‘Between work, childcare, and home duties—there is little time left over for me to take care of myself. I worry about making sure that everyone else is well taken care of that I often leave myself out.’” (69).
	Indigenous (*n* = 5)	“‘Yeah, but I was in really bad pain, but there was no way I could ring an ambulance because I had my three-year-old son, you know. So, I had to drive all the way to [locality] in this mega-pain, track down my friend and then be driven to the hospital.’ (#2)” Patient quote (83).
Immigrants & refugees (*n* = 9)	Non-White Hispanic (*n* = 3)	“‘That has been the most difficult part even when the pandemic started, I thought: I am going to die, I will never see [my children], I am medically fragile, what would happen if I have to be isolated, what would happen to my daughter, who would I leave her with, so many worries’ (IMW 10, 39 year old).” (88).
	Middle Eastern (*n* = 4)	“Several participants used volunteer doulas to accompany them to their delivery so their husbands could stay home with their other children; though grateful for this service, many participants said they would have preferred their husbands with them and their children safe in childcare.” (90).
	Asian (*n* = 2)	“Moving [cervical cancer] screening closer to the community would allow for older community members to come together socially, and for the younger women to assist each other with childcare.” (57).
Low-Income (*n* = 10)		“Decentralized resources made fulfilling medical and nonmedical needs challenging. In addition, many patients reported difficulty balancing other obligations, such as work and childcare, with prenatal services.” (34).
Unhoused (*n* = 3)		“Principal barriers for street outreach clients included personal–family issues, lack of insurance/Medicaid, ignorance, suspicion, and/or aversion to AOD treatment (methadone maintenance especially), ‘hassles’ with Medicaid, lack of personal ID, lack of ‘slots,’ limited access to intake, homelessness, childcare–child custody issues.” (67).
Geographically underserved (*n* = 4)	Rural (*n* = 1)	“In comparison, 69 respondents also identified challenges to attending in-person visits, including taking time off work (50.7%), childcare concerns (24.6%), access to transportation (7.2%), and money for gas (10.1%), as well as concern about exposure to SARS COV-2 through interaction with the healthcare system (7%).” (96).
	Urban (*n* = 1)	“‘If a lady does not have a good primary care doctor, it might be months before the person sees the doctor; they may not meet until the next scheduled visit, which would be 6 months…the mammogram information could be put in the person’s file and not be seen by the doctor until you get back to your next visit, which could be months later…also can get lost because the doctor does not find out if you have transportation, childcare, nor expresses the urgency of you getting to mammogram or other diagnosis; lack of follow up by primary care provider, everybody does not get sent all testing information. A patient can get lost in the 3–6 month follow up gap.’” (77).
	Global Health (*n* = 2)	“Women revealed leaving children with family and neighbors when possible, or if no caretakers were available, they were forced to take them along. As one woman reported, ‘Whenever we have to visit the District Hospital, it becomes difficult to leave our children alone or we request the neighbors. Sometimes they oblige, sometimes they do not.’” (49).
Substance use disorder (*n* = 8)		“In addition to challenges anyone with opioid use disorder may face—such as access to transportation, housing, and paying for treatment— previously incarcerated pregnant people are often hurdled with childcare needs, preparing for birth, fear of CPS, and difficulty securing stable housing due to drug offenses.” (84).

While reliance on social networks for childcare support was sometimes observed among minoritized communities (54), lack of social support was more often reported by caregivers as a main reason for facing unmet childcare needs (6, 31, 55, 61). Without social support for childcare, many caregivers also identified policies prohibiting bringing children to healthcare settings, further delaying their care (50, 92). Single mothers in New Zealand reported unmet childcare needs delayed emergency care, made appointments more stressful, made postpartum recovery difficult, and precluded one mother from accessing substance use treatment (84).

Additionally, literature identified that immigrants (*n* = 5) and refugees (*n* = 4) are likely to struggle with balancing healthcare access and childcare needs. Childcare needs identified in immigrant populations span across the globe, including immigrant Hispanic women in the United States (61, 75, 89), first-generation Somali women residing in London (55), and newly arrived immigrants from around the globe in Canada (58, 95). Refugee populations impacted by childcare needs included asylum seekers in the United States (100), refugees and asylum seekers in a government-funded housing center in Germany (105), and Syrian refugee women resettling in Canada (79, 91).

Among the 10 studies focused on low-income populations, paying for childcare or attending appointments was less prioritized over other competing financial demands like missing work (61, 70, 76). This experience of navigating changing constraints was also seen among postpartum mothers receiving care under Medicaid who lost insurance coverage shortly after birth (81). Other vulnerable populations identified in the literature included unhoused populations (*n* = 3), which were noted to have an added stressor related to custody of children and fear of being reported to Child and Family Services if children were brought to healthcare settings (47, 68). Two other studies examined childcare needs among unhoused caregivers seeking treatment for OUD (90) and clients of street outreach substance use disorder treatment programs (68).

Four studies focused on geographically vulnerable populations, including individuals living in rural areas (98, 110, 117) and urban medically underserved communities (78). Similarly, geographic isolation was explored in global health settings, such as among patients in Honduras (94). Alongside cost of care (94), inefficient medical resources (78), lack of social support (50), and a lack of access to technology (98), unmet childcare needs were an additional barrier that exacerbated spatial inequities.

Similarly, individuals with substance use disorders were noted to be a population susceptible to unmet childcare needs (*n* = 8), including the two studies previously mentioned exploring unhoused patients with addiction. Among patients with substance use disorders with childcare barriers, studies included female sex workers in northern Mexico with history of drug use (77), pregnant post-incarcerated women seeking treatment for OUD (85), among others (50, 89, 91, 100, 117). Individuals with substance use disorders often faced numerous barriers when seeking treatment, including financial difficulty, lack of insurance, and stigma, all factors compounded by extenuating childcare needs.

## 4 Discussion

This scoping review of childcare needs as a barrier to healthcare access identified five prominent themes from 91 inclusion studies: the role of childcare needs as a barrier to attending healthcare appointments; the opportunity for alternative care delivery models to circumvent childcare barriers; the impact of childcare needs on participation in medical research; the exacerbation of childcare needs during the COVID-19 pandemic; and the disproportionate impact of unmet childcare needs among marginalized populations. The data demonstrate that access to childcare is a SDOH that profoundly affects access to healthcare, from the ability to attend healthcare appointments to driving the transformation of care delivery models. While attention to childcare needs in the medical and public health literature is steadily increasing over time (Figure 2), additional investigation is needed to understand the mechanisms of childcare barriers in different disease states and practice settings and test interventions for childcare needs.

Our analysis demonstrated that the majority of studies on childcare needs in healthcare explore the role of childcare barriers on appointment completion and deferral of medical care (theme #1). Childcare barriers to attending healthcare appointments were represented across a variety of clinical contexts, unsurprisingly in women’s health settings like obstetric care and family planning, but also in the domains of substance use disorder treatment, cancer screening and treatment, and mental health. The breadth of relevance of childcare barriers across disease states and care settings suggests how widespread childcare needs are as a HRSN and how they may impact presentation to and engagement in healthcare through multiple heterogeneous mechanisms. Nonetheless, our analysis does find that childcare needs were often included in studies as one of multiple social determinants (*n* = 47, 51.6% of studies categorized in theme #1), rather than a discrete social need. Indeed, in screening of eligible studies, abstract texts and introductions of some studies referenced the term childcare but failed to study childcare needs as a variable or even allocate dedicated discussion to the role of childcare needs (*n* = 4). To truly characterize access to childcare as a SDOH, focused analysis is needed with dedicated attention to the role of childcare as a distinct driver of healthcare access. Importantly, current standardized tools to screen for HRSN do not proactively screen for childcare or caregiving needs (118–120); our findings would support incorporation of childcare needs in routine HRSN questionnaires to increase screening and ultimately inform intervention design.

Within theme #1’s focus on the impact of childcare barriers on timely appointment completion, theme #3 similarly found childcare barriers to participating in research. The theme of research participation is of unique interest given increasing attention to the need for diversification of medical research and clinical trials (121, 122), and disparities in clinical outcomes related to differential access to cutting-edge therapeutics (123, 124). All studies included in this theme intentionally acknowledged an understanding of childcare limiting research participation and facilitated childcare support to maximize recruitment, particularly for minoritized populations. Childcare needs commonly affect multiple aspects of research involvement, including limiting caregivers’ participation in both short-term focus group sessions and long-term clinical trials. While most research recruitment interventions addressed childcare needs with on-site childcare services, future work in this area may consider how alternative care delivery models, including community outreach methods, can improve access to research participation by reducing constraints from childcare needs.

Theme #2 highlighted the opportunity for care delivery redesign to circumvent the childcare barriers of themes #1 and #3. While possible solutions to attending in-person appointments explored in theme #1 included on-site childcare facilities, subsidizing childcare facilities, subsidizing childcare, or dedicated case management for childcare services, theme #2 highlighted the opportunity to develop new healthcare delivery systems obviating the need to travel to appointments altogether, namely through telehealth and home-based care models. This theme’s spotlight on the rise of telehealth intersects with theme #4 exploring the impact of COVID-19 on childcare needs, as pandemic conditions also galvanized and accelerated virtual care. While the COVID-19 pandemic affected many aspects of healthcare delivery and impacted the population at large, individuals with childcare needs experienced exponentiated difficulty accessing healthcare while balancing competing demands of social distancing, childcare responsibilities in the absence of schools and daycares, and their own health needs (125, 126). Consistent with prior literature exploring SDOH amidst pandemic conditions (127–129), theme #4 highlighted how the COVID-19 pandemic exacerbated and unmasked pre-existing inequities related to childcare responsibilities.

Theme #5 traversed across the preceding four themes, highlighting the intersection of childcare needs with other domains of social vulnerability. Unsurprisingly, studies suggested that individuals from marginalized backgrounds based on their race and ethnicity, income-level, or experiencing social risk factors (e.g., navigating homelessness, financial strain, recent immigration) are disproportionately affected by unmet childcare needs. Vulnerable populations experienced unique considerations in the experience of childcare needs including fear of losing custody of their children, institutional policies restricting bringing children to appointments, and competing financial demands including paying for childcare or taking time off work. Theme #5 points to structural inequities that belie childcare needs: gender inequity and misogyny, income inequality, systemic racism and xenophobia, among others. These structural factors lie upstream of intersecting SDOH that can amplify and compete with childcare needs: homelessness, food insecurity, transportation barriers, and financial insecurity (130–133). Structural interventions through childcare policy reform such as subsidies, tax credits, and expansion of Head Start and Early Head Start programs (19, 134) have the potential to alleviate childcare needs through social supports; such policy interventions have the potential for collateral benefits to other co-occurring SDOH.

This review collated sources utilizing heterogeneous methodologies, including qualitative analysis, survey methods, observational study design, and randomized controlled trials. Qualitative and survey methods predominated; this finding is consistent with the relative nascency of childcare needs as a research topic in the medical and public health literature, recognizing that open-ended and descriptive analyses are needed to guide future research questions. Even among randomized controlled trials in the inclusion studies (*n* = 3), childcare needs were explored as a variable affecting recruitment strategies or as a mediating factor, not as an exposure or outcome. Additional studies examining childcare needs as the primary exposure of interest are needed to establish clearer causal pathways between childcare needs and health outcomes. Existing literature remains at the descriptive base of the evidence hierarchy; future research should explore causal inference between childcare needs and health outcomes with more advanced methodologies, policy evaluation, and ultimately through intervention testing. Future research could unpack multiple potential mechanisms through which childcare needs affect healthcare access: appointment adherence, delayed or deferred care, competing HRSN, convenience of healthcare, and balancing childcare and self-care. In this review, barriers posed by childcare needs to attending appointments emerged as the most prominent causal mechanism identified, and these studies were most likely to recommend onsite childcare for future intervention testing.

This review revealed other important gaps in the literature. Notably, the vast majority of studies were conducted in developed countries in North America and Europe (*n* = 85, 93.4%). The experience of childcare needs in developing countries remains underrepresented in the current literature, and the five themes identified in our analysis may not generalize to under-resourced settings. For example, the relevance of childcare barriers to research participation (theme #3) may be of lower priority in lower-income countries, and alternative care delivery models through community outreach or self-care innovations may be more relevant than digital health interventions (theme #2). Theme #5, exploring the role of childcare needs alongside other measures of marginalization, would be particularly salient in these settings. Additional research in these countries is needed, especially given that the disproportionate childrearing responsibilities borne by women are implicated in gender inequities in developing countries (135).

Moreover, the vast majority of study populations among inclusion studies were women; 64.8% (*n* = 59) were comprised exclusively of women, and only 2 studies were comprised of a majority of men. This finding stresses the inequitable burden of caregiving responsibilities faced by women and the unique relevance of access to childcare as a SDOH among women. Nonetheless, the current literature may undervalue the childcare contributions made by men (136) and underestimate the role of childcare barriers faced by male caregivers; additional research is needed to capture these experiences. Holistic approaches, inclusive of diverse family structures, are needed to advance public health understanding of access to childcare as a SDOH.

Our review has key limitations. As a scoping review, the broad goal of this study in surveying multiple disciplines across diverse literature sources necessitated the inclusion of heterogeneous study designs. To query interdisciplinary sources of literature, we searched across databases, including outside healthcare sources, which may not have been completely comprehensive. The heterogeneous results of our interdisciplinary search make it difficult to systematically appraise the quality of studies for exclusion in our study criteria. Similarly, broad descriptions of childcare, consisting of variable ages, numbers of children, and types of relationships between child and caregiver, were accepted in surveying the literature since there was no standardized definition for childcare and our study team sought to define “caregiver” inclusively. Lastly, there may be studies published since June 1, 2023, the time the search was conducted, on this topic that were not included based on our criteria. It is important to note that the critical appraisal for access to childcare as a SDOH remains nascent in the literature. The purpose of this scoping review was to serve as a starting point for new lines of inquiry to understand access to childcare through the lens of SDOH. Strengths of this review include an inclusive search strategy that aggregated inclusion studies from diverse, interdisciplinary sources. Additionally, we opted for full-text reviews during our eligibility process in contrast to initial abstract screening typically performed in scoping reviews as we quickly learned that potentially eligible articles failed to mention childcare in the entirety of the abstract. This was a common finding that illustrates the cursory treatment of childcare needs alongside other barriers to healthcare access. Full-text source review enabled our team to assess the heterogeneity of childcare needs represented in the current literature.

As our current healthcare system moves toward structural changes to truly increase access to care, this review demonstrates growing evidence for the role of childcare needs as a driver of healthcare access. The role of childcare needs should be further explored as medicine and public health work to make healthcare more accessible to all populations. The relevance of childcare needs in women’s health and marginalized populations requires an intersectional approach, as highlighted in our study findings. As an underrecognized HRSN, childcare needs warrant adoption of screening to better understand the extent of childcare barriers in access to care and to inform development and implementation of interventions. Future research dedicated to access to childcare as a distinct, clinically significant SDOH is needed to improve equitable access to care.

## Data Availability

The raw data supporting the conclusions of this article will be made available by the authors, without undue reservation.
